# Stuttering in individuals with Down syndrome: a systematic review of earlier research

**DOI:** 10.3389/fpsyg.2023.1176743

**Published:** 2023-11-29

**Authors:** Silje Hokstad, Kari-Anne B. Næss

**Affiliations:** ^1^Department of Education, Lillehammer, Inland Norway University of Applied Sciences, Lillehammer, Norway; ^2^Department of Special Needs Education, University of Oslo, Oslo, Norway

**Keywords:** Down syndrome, stuttering, speech, disfluency, stuttering assessment, systematic review

## Abstract

The main objective of this systematic review was to synthesize the evidence on the occurrence and characteristics of stuttering in individuals with Down syndrome and thus contribute knowledge about stuttering in this population. Group studies reporting outcome measures of stuttering were included. Studies with participants who were preselected based on their fluency status were excluded. We searched the Eric, PsychInfo, Medline, Scopus, and Web of Science Core Collection databases on 3rd January 2022 and conducted supplementary searches of the reference lists of previous reviews and the studies included in the current review, as well as relevant speech and language journals. The included studies were coded in terms of information concerning sample characteristics, measurement approaches, and stuttering-related outcomes. The appraisal tool for cross-sectional studies (AXIS) was used to evaluate study quality. We identified 14 eligible studies, with a total of 1,833 participants (mean = 131.29, standard deviation = 227.85, median = 45.5) between 3 and 58 years of age. The estimated occurrence of stuttering ranged from 2.38 to 56%, which is substantially higher than the estimated prevalence (1%) of stuttering in the general population. The results also showed that stuttering severity most often was judged to be mild-to-moderate and that individuals with Down syndrome displayed secondary behaviors when these were measured. However, little attention has been paid to investigating the potential adverse effects of stuttering for individuals with Down syndrome. We judged the quality of the evidence to be moderate-to-low. The negative evaluation was mostly due to sampling limitations that decreased the representability and generalizability of the results. Based on the high occurrence of stuttering and the potential negative effects of this condition, individuals with Down syndrome who show signs of stuttering should be referred to a speech and language pathologist for an evaluation of their need for stuttering treatment.

## 1 Introduction

Stuttering is a speech-fluency disorder that involves the frequent and significant interruption of typical fluency and flow of speech, which can have negative effects on emotional, behavioral, and cognitive functioning from an early age (see, e.g., Craig et al., 2009; Briley et al., 2019; Guttormsen et al., 2021). One group that is reported to have a high occurrence of stuttering is individuals with Down syndrome (see, e.g., Kent and Vorperian, 2013). Due to language disorder (see, e.g., Martin et al., 2009; Næss et al., 2011), speech-sound disorder, and inappropriate prosody, speaking rate, and voice (see, e.g., Kent and Vorperian, 2013; Jones et al., 2019; Wilson et al., 2019; Loveall et al., 2021), an individual with Down syndrome typically have pervasive communication difficulties. Because stuttering may further interrupt their communication (see, e.g., Evans, 1977; Maessen et al., 2022), the identification of stuttering in this population is important in understanding the magnitude of their communication difficulties and supporting their communicative success.

Traditionally, in typically developing individuals, stuttering has mainly been operationalized and assessed based on behavioral factors (see, e.g., Tichenor and Yaruss, 2019). Examples of these factors include the type and number of disfluencies produced: audible symptoms that cause interruptions of speech, including repetitions of sounds (c-c-c-cat), syllables (ba-ba-ba-balloon), and one-syllable words (go-go-go); the prolongation of sounds (mmmilk); and blockages or stoppages of sounds (≠balloon). These audible symptoms are often accompanied by secondary behaviors caused by tension or the struggle to speak (i.e., visual symptoms, such as facial grimaces, blinking, or head nodding in an attempt to avoid stuttering; see, e.g., Bloodstein et al., 2021). Although there seems to be an agreement that speech behaviors are identifiers of stuttering, there is disagreement concerning which behaviors are symptomatic of stuttering, leading to differing operationalizations across studies (see, e.g., Einarsdottir and Ingham, 2005). One disagreement concerns whether word repetition is considered a stuttering disfluency. For example, in their operationalization of stuttering, Druker et al. (2020) excluded word repetitions, Millard et al. (2018) included word repetitions, and Boey et al. (2007) included only one-syllable word repetitions. Additionally, there have been various practices concerning the threshold at which speech disfluency is considered to be stuttering and, therefore, requires treatment. In a systematic review of stuttering-treatment studies by Sjøstrand et al. (2021), the frequency criterion (cutoff score) at treatment intake varied from no cutoff (Lewis et al., 2008) to a cutoff of a minimum of 3% of syllables stuttered (Harris et al., 2002; Lattermann et al., 2008). The use of frequency cutoff scores in the assessment of stuttering has been debated because (a) variability in stuttering across time and situations may cause participants to be wrongly classified if judgments are based on the percentage of stuttered syllables in only one speech sample (Constantino et al., 2016; Tichenor and Yaruss, 2021), (b) participants whose stuttering frequency is at the margins of the criterion set can be wrongly classified as non-stuttering (Tumanova et al., 2014), and (c) the adverse effects are not determined based on the frequency of overt speech disruptions (Koedoot et al., 2011; Blumgart et al., 2012), as the potential adverse effects of stuttering may also be critical for individuals with mild stuttering (i.e., mild based on listener evaluation; Beilby, 2014).

An increased awareness of the potential adverse effects of stuttering has led to a heightened focus on affective and cognitive reactions in the operationalization and assessment of stuttering. Affective reactions refer to feelings and emotions (e.g., feeling embarrassed, ashamed, or anxious), while cognitive reactions refer to a person’s thoughts (e.g., anticipation) and identity (e.g., low self-confidence or self-esteem; Tichenor and Yaruss, 2019). Assessment procedures that are solely based on listener evaluations of observable behaviors can therefore be criticized for not considering the multidimensionality of stuttering. Based on a multidimensional understanding, the stuttering assessment will preferably also involve an evaluation made by the individual who stutters. As stuttering behavior can be highly variable across time and contexts (Tichenor and Yaruss, 2021), a combination of assessment approaches and outcome measures may provide a holistic picture of the condition. Additionally, for individuals with Down syndrome, who often have limited expressive language skills and short verbal expressions (see, e.g., Berglund et al., 2001; Chapman and Hesketh, 2001; Zampini and D’Odorico, 2011), it may be a challenge to record speech samples of at least 200 words, which is typically recommended for speech evaluation (Ward, 2018). Thus, using a combination of assessment strategies and outcome measures seems especially important for this clinical group.

Several narrative reviews of research on stuttering in individuals with Down syndrome exist (Zisk and Bailer, 1967; Stansfield, 1988; Van Borsel and Tetnowski, 2007; Kent and Vorperian, 2013; Bloodstein et al., 2021). These reviews refer to disagreements in the field concerning whether individuals with Down syndrome display genuine stuttering. These arguments are related to the simultaneous presence of other speech and communication disorders, as well as a lack of evidence for these individuals’ secondary behaviors and awareness of their disfluency (Van Borsel and Tetnowski, 2007; Bloodstein et al., 2021). Challenges in the previous research literature have been highlighted. Operationalizations of stuttering are either not described or imprecisely described in several research reports (Zisk and Bailer, 1967; Kent and Vorperian, 2013), and the assessment procedures used in the typical population are not necessarily appropriate for individuals with disorders of intellectual development (Stansfield, 1988). Furthermore, several gaps in the research literature have been noted, such as limited knowledge concerning the presence or absence of secondary behaviors, the level of awareness and potential adverse effects of stuttering in this population (Zisk and Bailer, 1967; Van Borsel and Tetnowski, 2007), and whether stuttering is more common in male participants than female participants, as is suggested to be the case in the typical population (Van Borsel and Tetnowski, 2007). Additionally, Kent and Vorperian (2013) show a wide range in terms of participants’ age within studies. As studies of stuttering in the typical population have found that both the occurrence (Reilly et al., 2009, 2013) and the overt and adverse symptoms of stuttering may change with age (Guitar, 2014), samples with wide age ranges may bias the results. These abovementioned reviews have not used a systematic approach (see, e.g., Higgins et al., 2022), do not cover the last decade of research in the field, and have a broad scope (e.g., focusing on speech impairment in general; Zisk and Bailer, 1967; Kent and Vorperian, 2013), and are, therefore, somewhat superficial in their review of the scientific stuttering research literature. The highlighted challenges and gaps in our knowledge about stuttering in individuals with Down syndrome call for an updated review of the literature, including a more in-depth discussion about how stuttering is operationalized and assessed in this clinical group. In the current review, we therefore summarize, assess, and synthesize the relevant existing research literature on stuttering in individuals with Down syndrome. Considering the potential negative effects of stuttering, a comprehensive overview of the relevant research has the potential to bolster the development of better strategies with which to identify those who stutter and may need treatment. The following research questions led the review process:

1. How is stuttering operationalized and measured in the included studies?
2. What is the estimated occurrence of stuttering in the included studies?
3. Does the estimated occurrence of stuttering in the included studies vary according to gender and age?
4. What characterizes stuttering in individuals with Down syndrome based on the findings of the included studies?

## 2 Methods

This article has been registered in Prospero in advance, and the registration ID is CRD42021273799.

To answer the research questions, we conducted a systematic literature review using explicit, accountable methods in line with standards prescribed by Gough and Thomas (2016) and Higgins et al. (2022). The study-selection process is presented using the Preferred Reporting Items for Systematic Reviews and Meta-Analysis (PRISMA; Page et al., 2021).

All statistical analyzes were conducted in SPSS statistics. We evaluated the strength of inter-rater agreement in the study-selection process, data extraction, and quality analysis by calculating Cohens’s Kappa (κ; see, e.g., Gisev et al., 2013). Confidence intervals (95%) were calculated manually using the standard normal table (z-score table).

### 2.1 Eligibility criteria

In the current review, we included observational studies that reported at least one individual outcome measure of stuttering in individuals with Down syndrome. These could be studies that investigated stuttering via direct assessments or reports from a third party, such as parents or speech and language pathologists (SLPs). Studies in which the author(s) stated that they investigated stuttering were included. To answer the research questions, only studies that included occurrence estimates of stuttering (% and/or number) were eligible for inclusion. Thus, studies with samples that were preselected based on fluency status were excluded. Studies that investigated the co-existence of stuttering and other developmental speech disorders were included if the stuttering data were separated from other types of data. Mixed-etiology studies were considered for inclusion if they reported separate results for the participants with Down syndrome.

### 2.2 Search strategy

We developed the search strategy using words related to Down syndrome and stuttering. The search was conducted on 3rd January 2022 in the following databases: PsycINFO (Ovid interface, from 1806 onward), MEDLINE (Ovid interface, from 1946 onward), Eric (Ovid interface, from 1965 onward), Scopus (from 1960 onward), and Web of Science Core Collection (from 1945 onward). See Table 1 for the search strategy used in PsychINFO (Ovid interface, from 1806 onward). We verified and supplemented the electronic database search by searching (1) previous narrative reviews of stuttering or speech disfluency in individuals with Down syndrome and/or intellectual disability (Zisk and Bailer, 1967; Stansfield, 1988; Van Borsel and Tetnowski, 2007; Kent and Vorperian, 2013), (2) the reference lists of the included articles, and (3) acknowledged speech- and language-pathology journals and Google Scholar.

**Table 1:** Search strategy for PsychINFO.

Search strategy	
1. (Down* syndrome or Trisomy 21 or mongol*).mp. [mp = title, abstract, heading word, table of contents, key concepts, original title, tests and measures, mesh]	
2. (Stutter* or stammer* or disfluency* or non-fluency* or fluency disorder*).mp. [mp = title, abstract, heading word, table of contents, key concepts, original title, tests and measures, mesh]	
3. 1 and 2	

No restrictions were set with regard to publication date or language.

### 2.3 Study selection

The study-selection process had two phases. First, we screened the headings and abstracts and retrieved full-text sources that seemed to meet our inclusion criteria, as well as full-text sources that required further inspection. Second, we assessed the eligibility of these full-text sources. If a source appearing in our search was a chapter in an anthology, we read that specific chapter. When the source appearing in our search was a complete book, we first screened the index and then read the chapter(s) that were relevant to the topic of stuttering and/or disorders of intellectual development. See Appendix A for detailed information on the screening procedures.

#### 2.3.1 Screening of headings and abstracts

**Figure 1:**
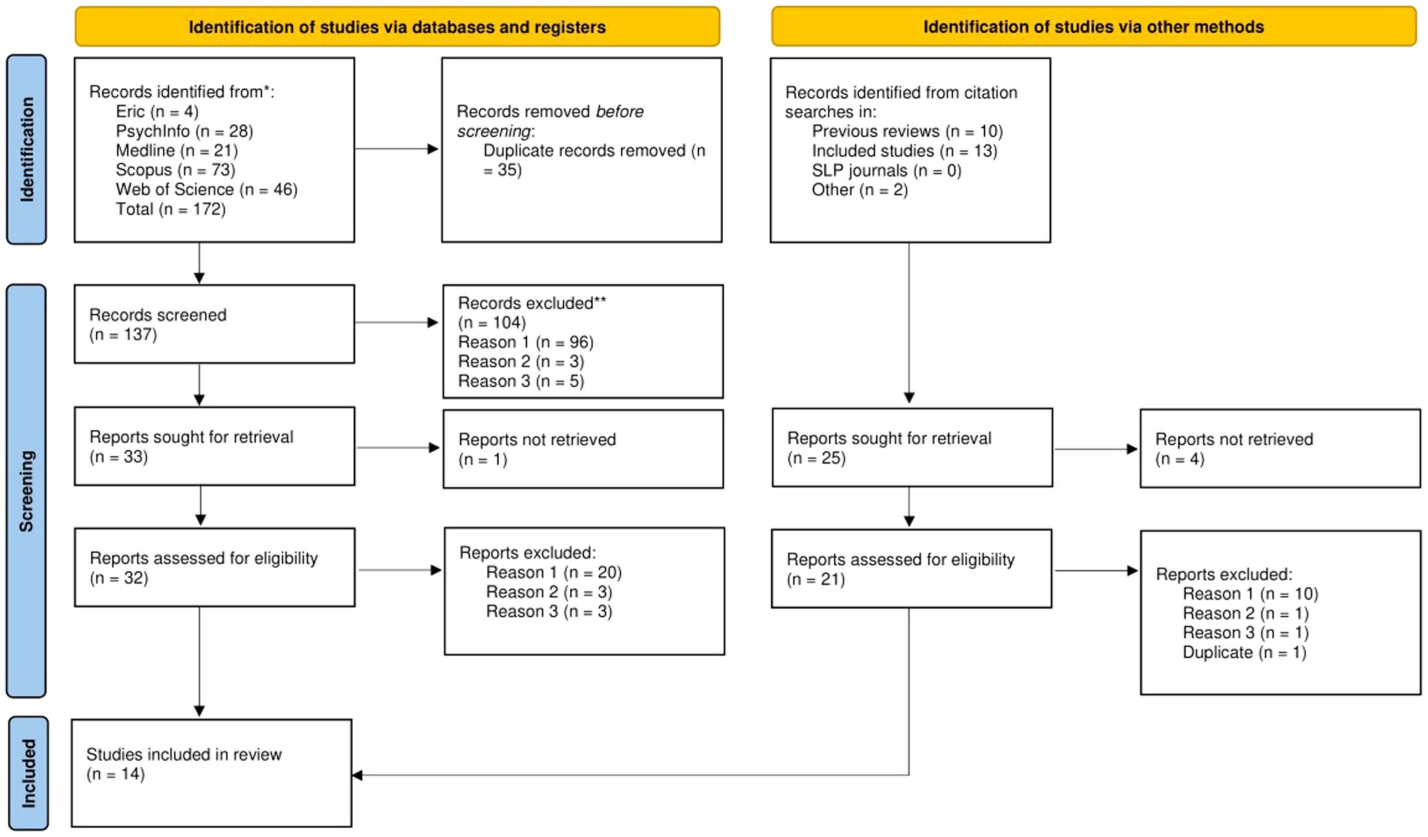
PRISMA flow diagram (Page et al., 2021).

The authors individually screened all titles and abstracts yielded by the systematic search against the eligibility criteria. Inter-rater reliability was calculated based on the agreement between the review authors regarding whether to include or exclude a study, as well as the reason for exclusion. There was good agreement between the review authors, κ = 0.715 (95% CI, 0.597 to 0.833), *p < 0*.001. Disagreements (n = 18 of 137 sources) were resolved through discussions between the review authors, including a reexamination of the headings and abstracts. In these discussions, the review authors were equal in status. Most disagreements concerned the reason for exclusion (i.e., not whether the study should be included). See Figure 1 (flow chart) for a record of the reasons for excluding sources in the heading- and abstract-screening phase.

#### 2.3.2 Assessment of full-text documents

A total of 33 sources were sought for retrieval based on the systematic search. Of these 33 sources, we were able to retrieve full-text manuscripts from 32. Additionally, we sought full-text manuscripts of potentially relevant sources located through supplementary searches (previous reviews, the reference lists of included articles, and free searches in relevant journals and Google Scholar). The supplementary searches revealed 25 sources, Of these 25 sources we were able to retrieve full-text manuscripts from 21. This included our own study (Hokstad et al., 2022), which was not yet published at the time of the search. See the description of the data-extraction process and quality analysis for information about how this study was treated in the review process. One study was excluded without further assessment due to beeing a duplicate.

A total of 52 sources were assessed against the eligibility criteria in the full-text assessment phase. Three sources were assessed in collaboration between the review authors for training purposes (Evans, 1977; Wilcox, 1988; Borsel and Vandermeulen, 2008). During this training, we first assessed the sources independently against the eligibility criteria before comparing and discussing our decisions. We also revised our eligibility criteria when these sources were found to be ambiguous. Our own study, Hokstad et al. (2022), was assessed by an independent third party, a trained speech and language pathologist and assistant professor at the University of Oslo. Three sources were published in languages not mastered by the review authors (Kehrer, 1973; Rabensteiner, 1975; Takagi and Ito, 2007). These sources were assessed in collaboration with third party evaluators a trained speech and language pathologist and assistant professor at the University of Oslo whose first language is German and a professor at Nagoya University whose first language is Japanese.

The remaining 45 sources were screened individually and in duplicate. Evaluations were based on the agreement between the review authors regarding whether to include or exclude a study, as well as the reason for exclusion. There was good agreement between the review authors, *κ* = 0.749 (95% CI, 0.580 to 0.918), *p < 0*.001. Disagreements (*n* = 7 of 45 sources) were discussed and resolved between the two review authors, who were equal in status. Four of the disagreements concerned whether a source met the inclusion criteria. The remaining three disagreements concerned the reason for exclusion. The disagreements were resolved through a reexamination of the text and, on one occasion, making contact with the main author of one study for clarification (Maessen et al., 2021). See Figure 1 (flow chart) for a record of the reasons for excluding studies in the full-text-screening phase and Appendix B for examples of the characteristics of the excluded sources on topics related to stuttering in individuals with Down syndrome.

### 2.4 Data extraction process

A total of 14 studies were eligible for inclusion. We extracted information related to sample characteristics, measurement approaches, and outcomes. We developed the coding scheme for the data extraction and discussed the content of each category. Then, we selected four sources (Preus, 1972; Devenny and Silverman, 1990; Stansfield, 1990; Salihovic et al., 2012) that we collaboratively assessed to refine our coding categories and training before double-coding. The training included the independent assessment of each source based on our understanding of the coding scheme. Next, we compared our results and discussed our differences. In cases in which we found our category descriptions to be ambiguous, we revised these descriptions. See Appendix C for the coding scheme for data extraction. One source published in a language not mastered by the review authors was coded in collaboration with a third party (Rabensteiner, 1975), a trained speech and language pathologist and assistant professor at the University of Oslo, whose first language is German. The study by Hokstad et al. (2022) was coded by an independent coder who is a trained speech and language pathologist and assistant professor at the University of Oslo. For the remaining eight eligible studies, the review authors extracted data independently and in duplicate. Disagreements were resolved through a reevaluation of the text and discussions between the review authors, who were equal in status. We evaluated the strength of inter-rater agreement by calculating Cohen’s kappa (κ) (see, e.g., Gisev et al., 2013) for each stuttering variable. The agreement between the review authors varied from good, *κ* = 0.724 (95% CI, 0.416 to 1.000), *p < 0*.001, to very good, *κ* = 1.000 (95% CI, 1.000, 1.000), *p < 0*.001. See Appendix D for κ values for each stuttering variable.

### 2.5 Quality appraisal

The appraisal tool for cross-sectional studies (AXIS) was used to assess the quality of the included studies. The tool includes items assessing sampling, justifications, clarity, and precision in descriptions of aims/objectives, methods, and results, as well as the reliability and validity of the measurement instruments (see Downes et al., 2016). For studies that included participants who did not have Down syndrome, we considered the information about the participants with Down syndrome only. In studies that investigated other areas of functioning, in addition to stuttering, we considered factors related to the measurement instruments and methodological transparency of the stuttering measures only (Q8–Q11). We made one adjustment when scoring the AXIS items; we only used the categories YES/NO (not using the “Do not know” category). Because scientific transparency is necessary for valid interpretations of the study results and the evaluation of research quality, negative evaluations were given when information in the study was lacking or insufficient for interpretation.

Again, one source (Rabensteiner, 1975) published in German, a language not mastered by the review authors, was coded in collaboration with a third party, and the authors’ own study (Hokstad et al., 2022) was coded by an independent judge. Before coding and double-coding, the authors discussed each item of appraisal and selected three sources for training purposes (Preus, 1972; Devenny and Silverman, 1990; Salihovic et al., 2012). The authors coded the remaining nine studies independently and in duplicate. Disagreements were resolved through reassessments of the articles in question and discussions between the review authors, who were equal in status. We evaluated the strength of the inter-rater agreement for the quality assessment by calculating Cohen’s kappa (see, e.g., Gisev et al., 2013). The inter-rater reliability was calculated based on the agreement between the review authors on each of the 20 AXIS items. Agreement varied from moderate, *κ* = 0.630 (95% CI, 0.297, 0.963), *p* < 0.001, to very good, *κ* = 1.000 (95% CI, 1.000, 1.000), *p* < 0.001. See Appendix E for the κ values for each AXIS item.

### 2.6 Data synthesis

We estimated the occurrence of stuttering in the total sample, per gender and per age group, by combining all samples included in the current review.

Occurrence = (the total number of individuals who stutter * 100)/total N.

In the data synthesis, we used the occurrence estimates reported in each individual study, independent of how stuttering was operationalized, thus combining different operationalizations of stuttering. Furthermore, we evaluated sample characteristics, measurement approaches, and stuttering outcomes by conducting a narrative synthesis of the findings consisting of statistical (frequencies, numeric summarizations, average calculations, and numeric comparisons) and narrative (content comparisons and grouping in overarching categories) analyzes. The results are presented in text and table format.

## 3 Results

### 3.1 Study selection

A total of 14 studies met the eligibility criteria for inclusion in the current review. One study was the authors’ own study (Hokstad et al., 2022), which was not yet published at the time of the systematic search. The remaining studies were identified through (1) a systematic search (*n* = 6), (2) a search of the reference lists of previous reviews (*n* = 6), and (3) the reference lists of included studies (*n* = 1). See Figure 1 for a flow chart depicting the selection process.

### 3.2 Study characteristics

The included studies were published between 1955 and 2022 and, as such, represent seven decades of research on stuttering in individuals with Down syndrome. However, most of the studies are older, with a majority (*n* = 10) having been published before 2000.

### 3.3 Operationalization of stuttering

Four studies did not contain any operationalization of stuttering (Gottsleben, 1955; Rabensteiner, 1975; Kumin, 1994; Schieve et al., 2009). In eight studies, stuttering was operationalized based on indicators related to speech behaviors alone (Rohovsky, 1965; Eggers and Van Eerdenbrugh, 2018; Hokstad et al., 2022) or in combination with secondary behaviors (Schlanger and Gottsleben, 1957; Martyn et al., 1969; Keane, 1970; Preus, 1972; Devenny and Silverman, 1990) and on affective and cognitive reactions to stuttering (Martyn et al., 1969; Keane, 1970; Preus, 1972). See Table 2 for an overview of the indicators included in the operationalization of stuttering across studies.

**Table 2:** Operationalization of stuttering.

Indicators	Studies													
	1	2	3	4	5	6	7	8	9	10	11	12	13	14
Speech behaviors^a^	–	–	–	–	–	–	–	–	–	–	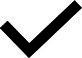	–	–	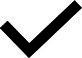
Repetitions^b^	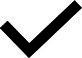	–	–	–	–	–	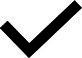	–	–	–	–	–	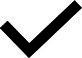	–
Part-word repetitions^c^	–	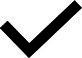	–	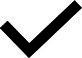	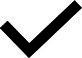	–	–	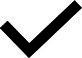	–	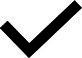	–	–	–	–
Single-syllable word repetitions	–	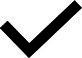	–	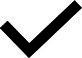	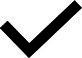	–	–	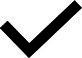	–	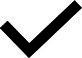	–	–	–	–
Multisyllabic whole-word repetitions	–	–	–	–	–	–	–	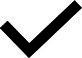	–	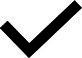	–	–	–	–
Prolongations	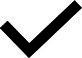	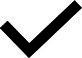	–	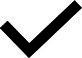	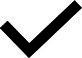	–	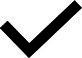	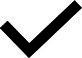	–	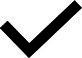	–	–	–	–
Blocks^d^	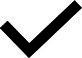	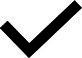	–	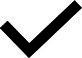	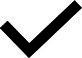	–	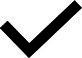	–	–	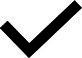	–	–	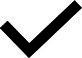	–
Secondary behaviors	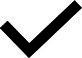	–	–	–	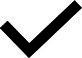	–	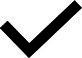	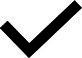	–	–	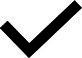	–	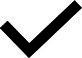	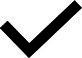
Cognitive reactions	–	–	–	–	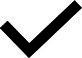	–	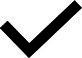	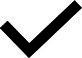	–	–	–	–	–	–
Affective reactions	–	–	–	–	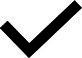	–	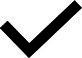	–	–	–	–	–	–	–

1 = Devenny and Silverman (1990), 2 = Eggers and Van Eerdenbrugh (2018), 3 = Gottsleben (1955), 4 = Hokstad et al. (2022), 5 = Keane (1970), 6 = Kumin (1994), 7 = Martyn et al. (1969), 8 = Preus (1972), 9 = Rabensteiner (1975), 10 = Rohovsky (1965), 11 = Salihovic et al. (2012), 12 = Schieve et al. (2009), 13 = Schlanger and Gottsleben (1957), 14 = Stansfield (1990). ^a^Speech behaviors not specified. ^b^Types of repetitions not specified. ^c^Includes the repetition of sounds and the repetition of syllables. ^d^Eggers and Van Eerdenbrugh (2018) report broken words (blocks within words) separately.

In addition to the presence of indicators of stuttering, four studies also reported the threshold at which (e.g., % syllables stuttered cutoff score) stuttering behaviors were considered clinically significant (Keane, 1970; Preus, 1972; Eggers and Van Eerdenbrugh, 2018; Hokstad et al., 2022). See Table 3 for an overview of the frequency cutoff scores used in these four studies. Finally, two studies (Stansfield, 1990; Salihovic et al., 2012) operationalized stuttering based on the frequency of stuttering disfluencies, the duration of stuttering blocks, and the number of physical concomitants (i.e., secondary behaviors), without specifying which types of disfluencies were considered and at what threshold disfluencies were considered stuttering. See Appendix F for a detailed overview of the operationalizations of stuttering in the included studies.

**Table 3:** Frequency cutoff scores.

Study	Cutoff	Unit of analysis
Eggers and Van Eerdenbrugh (2018)	3 or more	Per 100 syllables or a maximum number of syllables
Hokstad et al. (2022)	3 or more	Per total number of syllables
Keane (1970)	3 or more	Per total number of words
Preus (1972)	5 or more	Per 100 words

### 3.4 Measurement approaches

In two studies, stuttering was assessed indirectly through parental reports (Kumin, 1994; Schieve et al., 2009). As the parents simply reported whether their child stuttered or not, these studies provided limited information about stuttering besides the stuttering occurrence estimate.

In 12 studies, stuttering was identified through clinical judgment by either SLPs/SLP students and/or the researcher(s) themselves (Gottsleben, 1955; Schlanger and Gottsleben, 1957; Rohovsky, 1965; Martyn et al., 1969; Keane, 1970; Preus, 1972; Rabensteiner, 1975; Devenny and Silverman, 1990; Stansfield, 1990; Salihovic et al., 2012; Eggers and Van Eerdenbrugh, 2018; Hokstad et al., 2022). In eight of these 12 studies, stuttering was identified through speech-sample analysis (Rohovsky, 1965; Keane, 1970; Preus, 1972; Devenny and Silverman, 1990; Stansfield, 1990; Salihovic et al., 2012; Eggers and Van Eerdenbrugh, 2018; Hokstad et al., 2022), while in four studies, stuttering was identified through either written sources (Gottsleben, 1955; Schlanger and Gottsleben, 1957) or real-time observation (Martyn et al., 1969; Rabensteiner, 1975). Spontaneous speech samples were commonly elicited through planned speaking situations, such as play sessions (Eggers and Van Eerdenbrugh, 2018), conversations about pictures (Preus, 1972; Salihovic et al., 2012), and story retelling (Rohovsky, 1965; Hokstad et al., 2022). In six studies, audio data were collected (Rohovsky, 1965; Preus, 1972; Stansfield, 1990; Salihovic et al., 2012; Eggers and Van Eerdenbrugh, 2018; Hokstad et al., 2022), while video data were collected in two studies (Keane, 1970; Devenny and Silverman, 1990). The length of the speech samples and the amount of speech material collected varied greatly across studies; however, the speech samples were, with one exception (Hokstad et al., 2022), retrieved from only one speaking situation. Furthermore, while some studies used speech samples with variable lengths and amounts of speech (Preus, 1972; Hokstad et al., 2022), others based their evaluation on a set speech-sample length (Rohovsky, 1965; Keane, 1970; Stansfield, 1990) or a set number of words or syllables (Preus, 1972; Devenny and Silverman, 1990; Salihovic et al., 2012; Eggers and Van Eerdenbrugh, 2018). See Table 4 for a detailed overview of the measurement approach(es) used in each study.

**Table 4:** Measurement approaches.

Study	Assessor(s)	Instrument		
	Parent(s), SLP(s), author(s)/researcher(s), student(s), stutterer, or other	Clinical judgment, parental judgment, self-report, or other	Speaking situation as described in study	Speech sample (audio/video, duration and number of utterances, words, or syllables), written sources, real-time observation (duration and/or number of utterances, words, or syllables), or own experience
Devenny and Silverman (1990)	SLPs	Clinical judgment	Conversation about work and recreation	Speech sample (video, 10 min, first 150 words)
Eggers and Van Eerdenbrugh (2018)	Authors	Clinical judgment	Play session with toy or book adapted to age and interests	Speech sample (audio, 15 min, 50 utterances^1^)
Gottsleben (1955)	Author and SLPs	Clinical judgment	NR	Written sources
Hokstad et al. (2022)	Researchers	Clinical judgment	Picture book dialog and story-retelling	Speech sample (audio, unknown duration/number of utterances/words/ syllables)
Keane (1970)	1 = SLP 2 = SLPs and other (clinical experience with stutterers)	Clinical judgment	1 and 2 = Interviews about daily life and interests	1 = Real-time observation (*ca.* 10 min), 2 = Speech sample (video, mean duration 10 min)
Kumin (1994)	Parent	Parental judgment	NR	Real-time observation (NR)
Martyn et al. (1969)	SLPs and students	Clinical judgment	Conversation, interview, or reading sample adapted to the level of intellectual disability	Real-time observation (NR)
Preus (1972)	(1) NR (2) Other (personnel day institutions) (3) NR	Clinical judgment	(1) Spontaneous speech evoked by means of conversation pictures (2) Daily interaction (3) NR	(1) Speech sample (audio, mean duration 9.47 min, min/max = 3.5–28 min, minimum 200 words) (2) Real-time observation (NR) (3) NR
Rabensteiner (1975)	Other (two observers)	Clinical judgment	Test situation	Real-time observation
Rohovsky (1965)	10 grad. Students (speech and hearing science)	Clinical judgment	Story retelling	Speech sample (audio, 30 s)
Salihovic et al. (2012)	SLPs	Clinical judgment	Spontaneous speech elicited through pictures	1 and 2 = Speech sample (audio, minimum 200 syllables) 3 = real-time observation (minimum 200 syllables)
Schieve et al. (2009)	Adult family member (usually parent)	Parental judgment	NR	Real-time observation (NR)
Schlanger and Gottsleben (1957)	Authors/researchers	Clinical judgment	NR	Written sources
Stansfield (1990)	1 = Other (nursing or ATC staff) 2 and 3 = SLP and students	1 = Other (paid caregivers) 2 and 3 = Clinical judgment	1 = NR 2 = Informal interaction 3 = Assessment situation	1 = Real-time observation (NR) 2 = Real-time observation (5 min) 3 = Speech sample (audio, 30 min)

In cases where assessments have been conducted in several stages, each stage is numbered, SLP, speech and language pathologist; min, minutes; NR, not reported. ^1^When 50 utterances were not available, the maximum number of utterances was used. In cases in which assessments have been conducted in several stages, each stage is numbered, SLP, speech and language pathologist; ATC staff, adult training center staff; min, minutes; NR, not reported; grad, graduate.

### 3.5 Sample characteristics

A total of 1,833 (*M* = 131.29, SD = 227.85, median = 45.5) individuals with Down syndrome participated in the included studies, with sample sizes ranging from 26 to 897 participants. Kumin (1994) represented an extreme value (± 3 standard deviations from the mean) with 897 participants. The average sample size with this extreme outlier excluded was 72 participants (SD = 60.46, min = 26, max = 200). Participants of all ages were represented across the included studies. The studies that reported participant age had wide age ranges (Gottsleben, 1955; Rohovsky, 1965; Keane, 1970; Preus, 1972; Rabensteiner, 1975; Devenny and Silverman, 1990; Schieve et al., 2009; Salihovic et al., 2012; Eggers and Van Eerdenbrugh, 2018; Hokstad et al., 2022), with an average age gap of 21 years between the youngest and oldest participants. Hokstad et al. (2022) had the narrowest age spread, at 2 years, while Gottsleben (1955) had the widest age spread, at 43 years. Eight studies reported the gender distribution of the participants (Gottsleben, 1955; Rohovsky, 1965; Keane, 1970; Preus, 1972; Devenny and Silverman, 1990; Schieve et al., 2009; Eggers and Van Eerdenbrugh, 2018; Hokstad et al., 2022), and a total of 310 male and 278 female participants took part in these studies. Seven studies reported including only individuals who used speech (Gottsleben, 1955; Rohovsky, 1965; Preus, 1972; Devenny and Silverman, 1990; Kumin, 1994; Eggers and Van Eerdenbrugh, 2018; Hokstad et al., 2022). However, the level of speech proficiency is often not specified. See Table 5 for a detailed overview of the sample characteristics.

**Table 5:** Sample characteristics.

Study	Nationality	Sample size (N)	Gender		Age			Language proficiency
			Male	Female	M	Min	Max	
Devenny and Silverman (1990)	United States	31	20	11	41	30	58	Used speech^1^
Eggers and Van Eerdenbrugh (2018)	Belgium	26	12	14	8	3	12	Used speech^2^
Gottsleben (1955)	United States	36	23	13	27	9	52	Used speech^2^
Hokstad et al. (2022)	Norway	75	40	35	7	6	8	Used speech^2^
Keane (1970)	United States	200	100	100	15	6	46	Participants did and did not use speech^2^
Kumin (1994)	United States	897	NR	NR	NR	NR	NR	Used speech^2^
Martyn et al. (1969)	United States	42	NR	NR	NR	NR	NR	NR
Preus (1972)	Norway	47	21	26	NR	7	48	Speech intelligibility^3^
Rabensteiner (1975)	Germany	49	NR	NR	NR	5	15	Good receptive language skills^2^
Rohovsky (1965)	United States	27	11	16	15	9	20	Used three-word utterances or more
Salihovic et al. (2012)	Bosnia-Herzegovina	37	NR	NR	NR	6	17	NR
Schieve et al. (2009)	United States	146	83	63	NR	3	17	NR
Schlanger and Gottsleben (1957)	United States	44	NR	NR	NR	NR	NR	All but one participant used speech^2^
Stansfield (1990)	Scotland	176	NR	NR	NR	NR	NR	NR

NR, not reported; Min, minimum; Max, maximum. ^1^Indicated by verbal IQ of 43–77. ^2^As stated in the study. ^3^Judges able to understand half of the responses.

### 3.6 The occurrence of stuttering in individuals with Down syndrome

The reported occurrence of stuttering varied between 2.38 and 56.00% across the included studies. Combining all samples (total number of individuals who stutter * 100/total N) resulted in an occurrence estimate of 19.80%. Occurrence by gender or the information necessary to calculate occurrence by gender was reported in five studies (Gottsleben, 1955; Rohovsky, 1965; Keane, 1970; Devenny and Silverman, 1990; Eggers and Van Eerdenbrugh, 2018). The results suggest a gender factor of 2:1; 27.11% (45 of 166) of the male participants and 13.64% (21 of 154) of the female participants in these studies were determined to stutter. Information about the age of the stuttering participants was reported in six studies, and their ages ranged from 5 to 58 years of age (Gottsleben, 1955; Rohovsky, 1965; Keane, 1970; Stansfield, 1990; Salihovic et al., 2012; Eggers and Van Eerdenbrugh, 2018). When categorizing the included studies based on age groups (preschool age, school age, adulthood, and mixed), we found seven studies reported on mixed-age samples: from preschool age through adulthood (Kumin, 1994), preschool age through school age (Schieve et al., 2009; Eggers and Van Eerdenbrugh, 2018), and school age through adulthood (Gottsleben, 1955; Keane, 1970; Preus, 1972; Stansfield, 1990). Three of these studies reported separate findings based on age or age intervals, but the participants were not equally distributed across age groups (Stansfield, 1990; Kumin, 1994; Eggers and Van Eerdenbrugh, 2018). Three studies reported on only school-aged participants (Rohovsky, 1965; Salihovic et al., 2012; Hokstad et al., 2022), while one study reported on only adults (Devenny and Silverman, 1990). The remaining three studies could not be categorized due to the lack of information on participant age (Schlanger and Gottsleben, 1957; Martyn et al., 1969; Rabensteiner, 1975). See Table 6 for detailed information on the occurrence of stuttering across studies. See Table 7 for an overview of the occurrence of stuttering across age groups.

**Table 6:** Occurence of stuttering.

Study	Occurrence		Occurrence male/female		Occurrence by age group	Age of stutterers	
	%	*n*	%	*n*		*M*	Min/Max
Devenny and Silverman (1990)	42.00	13	45.00/36.00	9/4	Adults 13/31	NR	NR
Eggers and Van Eerdenbrugh (2018)	31.00	8	50.00/14.00	6/2	Pre-schoolers 1/4 School-age 7/22	10	5/13
Gottsleben (1955)	33.00	12	44.00/15.00	10/2	Mixed 12/36	NR	16/43
Hokstad et al. (2022)	53.34	40	NR	NR	School-age 40/75	NR	NR
Keane (1970)	10.00	20	16.00/4.00	16/4	Mixed 20/200	18	6/32
Kumin (1994)	17.00	153	NR	NR	Pre-schoolers 4/191 School-age 115/561 Adults 34/145	NR	NR
Martyn et al. (1969)	2.38	1	NR	NR	NR	NR	NR
Preus (1972)	40.95	19	NR	NR	Mixed 14/47	NR	NR
Rabensteiner (1975)	33.33	11	NR	10/1	NR	NR	NR
Rohovsky (1965)	48.00	13	36.36/56.25	4/9	School-age 13/27	15	9/19
Salihovic et al. (2012)	13.51	5	NR	NR	School-age 5/37	14	10/17
Schieve et al. (2009)	15.60	27	NR	NR	Mixed 27/146	NR	NR
Schlanger and Gottsleben (1957)	45.45	20	NR	NR	NR	NR	NR
Stansfield (1990)	11.93	21	NR	13/8	School-age: 1/NR Adults: 19/NR Missing: 1^1^	33	17/61

^1^In Stansfield (1990), detailed information about one of the stuttering participants with Down syndrome is missing.

**Table 7:** Occurrence by age group.

Age group	Studies	Participants	Occurrence of stuttering	
			*N*	%
Mixed samples	4	429	78	18.01
Adulthood	2	176	47	26.70
School age	6	771	191	24.77
Preschool age	2	195	5	2.56
NR	3			

*N* = number of participants. Studies that reported separate findings based on age or age intervals (Stansfield, 1990; Kumin, 1994; Eggers and Van Eerdenbrugh, 2018) are represented within more than one category. Each participant is represented once.

### 3.7 Stuttering outcomes

Stuttering frequency in individuals who stuttered was reported in four studies (Keane, 1970; Salihovic et al., 2012; Eggers and Van Eerdenbrugh, 2018; Hokstad et al., 2022). The approaches to calculating stuttering frequency varied, and these studies were, therefore, not directly comparable. For example, while Eggers and Van Eerdenbrugh (2018) reported an average of 5.1% stuttering-like disfluencies per 100 syllables, Keane (1970) reported an average of 11.35% stuttered words. The distribution of disfluency types in individuals who stutter was only reported in the dissertation by Keane (1970). Based on her reporting of stuttering types, 78% of the disfluencies in individuals with Down syndrome who stuttered (*n* = 20) were prolongations, which occurred in 19 of 20 participants, and 22% were part-word repetitions, which occurred in 17 of 20 participants.

Stuttering severity was reported in six studies. In half of these studies (Stansfield, 1990; Salihovic et al., 2012; Eggers and Van Eerdenbrugh, 2018), judgments were based on the total score on the Stuttering Severity Instrument (Riley, 1980, 1994), while in the other half (Rohovsky, 1965; Keane, 1970; Hokstad et al., 2022), judgments were based on placement on a severity scale after a perceptual evaluation. According to the findings of these studies, most of the participants displayed mild-to-moderate stuttering, and severe stuttering was rare.

Secondary behaviors were reported in six studies (Rohovsky, 1965; Keane, 1970; Preus, 1972; Stansfield, 1990; Salihovic et al., 2012; Eggers and Van Eerdenbrugh, 2018). Four of these studies reported the number of stuttering participants displaying secondary behaviors (Rohovsky, 1965; Keane, 1970; Stansfield, 1990; Eggers and Van Eerdenbrugh, 2018), while for the remaining two studies, this information was not reported (Salihovic et al., 2012) or was unclear (Preus, 1972). Across the studies that did report the occurrence of secondary behaviors, these behaviors were observed in 66.13% of the stuttering participants.

Outcomes related to the potential adverse effects of stuttering were reported in three studies (Martyn et al., 1969; Keane, 1970; Preus, 1972), all of which concluded that there was no evidence of affective or cognitive reactions in their participants. However, it must be noted that this is our interpretation based on the descriptive information that exists in these research reports. Martyn et al. (1969) stated that stuttering, in their participants, did not appear to be associated with anticipation or avoidance, while Preus (1972) stated that, even though there were signs of avoidance and postponement in some participants, none of them seemed to be aware of or embarrassed by their stuttering. Finally, Keane (1970) placed all stuttering individuals in one of Bloodstein’s four developmental phases of stuttering (Bloodstein, 1960) and reported that none of the participants had reached phase four, advanced stuttering, which includes the anticipation of stuttering, word substitutions, and avoidance of speaking, as well as evidence of fear and embarrassment. None of the more recent studies included information on affective or cognitive reactions to stuttering. See Table 8 for the stuttering outcomes and information regarding the instruments these outcomes are based on.

**Table 8:** Stuttering outcomes.

Study	Stuttering behavior				Adverse impacts	
	Total frequency	Frequency per disfluency type	Severity	Secondary behavior	Affective reactions	Cognitive reactions
Devenny and Silverman (1990)	NR	NR	NR	NR	NR	NR
Eggers and Van Eerdenbrugh (2018)	%SLD^a^ M 5.1 min/max 3–11 SSI score^b^ M 10	NR	n per category ^c^ Mild 5, Moderate 3 SSI score ^c^ M 18.38 min/max 12–26	Participants^d^ 6 of 8 SSI score^d^ M 3.8	NR	NR
Gottsleben (1955)	NR	NR	NR	NR	NR	NR
Hokstad et al. (2022)	%SLD ^e^ M 9.50 min/max 3.22–29.37	NR	Severity rating^f^ M 2.30 min/max 0.50–6.50	NR	NR	NR
Keane (1970)	%SLD^g^ per minute M 7.51 total M 11.35	Relative frequency part-word repetitions 22% prolongations 78% Participants with part-word repetitions 17/20 prolongations 19/20	n per category Very mild/Mild 1, Mild 2, Mild/Moderate 10, Moderate 5, Moderate/Severe 2	Participants 18 of 20	No affective reactions^h^	No cognitive reactions^h^
Kumin (1994)	NR	NR	NR	NR	NR	NR
Martyn et al. (1969)	NR	NR	NR	NR	NR	No cognitive reactions
Preus (1972)	NR	NR	NR	NR*	No affective reactions	No cognitive reactions
Rabensteiner (1975)	NR	NR	NR	NR	NR	NR
Rohovsky (1965)	NR	NR	n per category^a^ Mild 7, Moderate 6 Severity rating^a^ M = 1.5 Min/max = NR	Participants 5 of 13	NR	NR
Salihovic et al. (2012)	SSI score M 11.60 min/max 8–16	NR	SSI score M 26^b^ min/max 17–49	SSI score M 6.40 min/max 3–12	NR	NR
Schieve et al. (2009)	NR	NR	NR	NR	NR	NR
Schlanger and Gottsleben (1957)	NR	NR	NR	NR	NR	NR
Stansfield (1990)	NR	NR	n per category Mild 7, Moderate 7, Severe 6, Missing 1	SSI score NR Participants 12 of 21 Missing 1^c^	NR	NR

%SLD, percentage of syllables stuttered; SSI, stuttering severity instrument (Riley, 1994). ^a^Percent syllables stuttered per 100 syllables or maximum number of syllables when 100 syllables were not available. ^b^Based on the frequency score from the SSI (Riley, 1994). ^c^Based on the total score from the SSI (Riley, 1994). ^d^Based on the physical concomitant score from the SSI (Riley, 1994). ^e^Percentage of syllables stuttered per maximum number of syllables in each speech sample. ^f^Based on the stuttering severity rating (0–9) on the Stuttering Severity Rating Scale (Onslow et al., 2020). ^g^Percentage of stuttered words. ^h^Based on placement in one of Bloodstein’s four developmental stages of stuttering (Bloodstein, 1960). SSI, stuttering severity instrument (Riley, 1980, 1994). ^a^Based on stuttering severity rating on a scale from 0 to 5. ^b^Equals moderate degree of stuttering. ^c^In Stansfield (1990), detailed information about one of the stuttering participants with Down syndrome is missing.

### 3.8 Quality of the included studies

#### 3.8.1 Inter-rater reliability

Inter-rater reliability was given for some or all stuttering measures reported in three of the included studies (Keane, 1970; Eggers and Van Eerdenbrugh, 2018; Hokstad et al., 2022). See Table 9 for an overview of inter-rater reliability measures. Based on the low number of stuttering outcome measures tested for consistency across studies, test validity is an area of great insecurity in existing research on stuttering in individuals with Down syndrome. Additionally, research on typically developing individuals has raised concerns about the inter-rater reliability of both the Stuttering Severity Instrument (SSI; Davidow, 2021) and disfluency-type measures (Cordes, 2000; Einarsdottir and Ingham, 2005), both of which have been used across studies in the current review. It is therefore important, especially with this population, which has profound speech and language difficulties (see, e.g., Martin et al., 2009; Næss et al., 2011; Jones et al., 2019; Wilson et al., 2019; Loveall et al., 2021), that speech evaluations are performed by more than one rater.

**Table 9:** Inter-rater reliability.

Study	Variable	Reliability
Devenny and Silverman (1990)	Occurrence	NR
Eggers and Van Eerdenbrugh (2018)	%SLD^a^ SSI frequency score SSI physical Concomitant score SSI Total score (severity)	Agreement index percentage = 0.91^b^ NR NR NR NR
Gottsleben (1955)	Occurrence	NR
Hokstad et al. (2022)	%SLD^a^ Severity rating^a^	Percent agreement = 89.07 Percent agreement = 93.75^c^
Keane (1970)	%SLD^a^ Severity rating Secondary reactions Affective reactions Cognitive reactions	Pearson’s product moment Correlation coefficient = 0.970 NR NR NR NR
Kumin (1994)	Occurrence	NR
Martyn et al. (1969)	Occurrence Cognitive reactions	NR NR
Preus (1972)	Occurrence	NR
Rabensteiner (1975)	Occurrence	NR
Rohovsky (1965)	Occurrence Severity rating Secondary behavior	NR NR NR
Salihovic et al. (2012)	SSI total score^a^ SSI frequency score SSI physical Concomitant score	NR NR NR
Schieve et al. (2009)	Occurrence	NR
Schlanger and Gottsleben (1957)	Occurrence	NR
Stansfield (1990)	SSI total score^a^ SSI physical Concomitant score	NR NR

^a^Basis for occurrence estimate. ^b^‘Agreement index’ percentage (i.e., the number of agreements divided by the sum of agreements and disagreements regarding all disfluencies). ^c^Scores that were within one scale point of one another were judged to indicate agreement between the raters.

#### 3.8.2 Study quality appraisal

The studies in the current review met between 6 and 19 of 20 potential criteria items of the AXIS tool (*M* = 11.07, SD = 3.99). Thus, the results of the quality appraisal indicate that the quality of the included studies ranges from low to high, with most studies being of moderate-to-low quality. Many studies were found to be lacking in areas related to sampling procedures. First, most studies had small sample sizes, and all studies lacked sample size justifications (AXIS item 3). To provide an example regarding the number of participants necessary for an accurate estimation of stuttering frequency in a population, given an estimated population of 3,725 individuals with Down syndrome in Norway (De Graaf et al., 2021), the minimum sample size for determining the frequency of stuttering in the Norwegian population is 349 participants (95% confidence level, anticipated frequency unknown; Dean et al., 2013). Additionally, as the incidence of stuttering is known to be influenced by the age group sampled (Reilly et al., 2009, 2013), the appropriate sample size is likely to be even higher. Second, convenience sampling was common across studies, and only two studies reported systematic recruitment procedures (Schieve et al., 2009; Hokstad et al., 2022). Thus, for most of the included studies, it was unlikely that samples closely represented the population of individuals with Down syndrome they were drawn from (AXIS items 5 and 6). Small samples recruited through convenience sampling are not well suited to providing estimates of occurrences, as random sampling and adequate sample sizes are necessary for precise prevalence and incidence estimates (Munn et al., 2014). Inaccuracies in the occurrence estimates and related outcomes must therefore be assumed. A related area of concern is the treatment and reporting of non-responders (AXIS items 7, 13, and 14), which is unreported or unclear in several studies. Given the large variation in speech and language proficiency in this population (Karmiloff-Smith et al., 2016), it is, for example, likely that some participants across studies did not provide sufficient speech and/or intelligible speech for an evaluation of stuttering to be conducted. However, in many of the included studies, it is generally not clear whether and how many participants were lost due to restricted speech and language. See Table 10 for an overview of the quality assessment of the included studies for each AXIS item.

**Table 10:** Quality appraisal.

Study	AXIS item																				Total score per study
	1	2	3	4	5	6	7	8	9	10	11	12	13	14	15	16	17	18	19	20	
Devenny and Silverman (1990)																					7
Eggers and Van Eerdenbrugh (2018)																					13
Gottsleben (1955)																					12
Hokstad et al. (2022)																					19
Keane (1970)																					14
Kumin (1994)																					6
Martyn et al. (1969)																					10
Preus (1972)																					10
Rabensteiner (1975)																					7
Rohovsky (1965)																					8
Salihovic et al. (2012)																					7
Schieve et al. (2009)																					15
Schlanger and Gottsleben (1957)																					16
Stansfield (1990)																					9
Total score per item	14	12	0	14	3	3	5	10	9	11	7	6	3	4	11	7	12	6	14	2	

The AXIS critical appraisal tool for cross-sectional studies (Downes et al., 2016) was used to assess study quality. Green indicates an item score of 1 (i.e., a positive evaluation). Red indicates an item score of 0 (i.e., a negative evaluation).

## 4 Discussion

The current review has five main findings: (1) there was no common approach to identifying stuttering in individuals with Down syndrome, but there was a one-sided focus on observational aspects; (2) the occurrence estimates were generally high but varied across studies; (3) the occurrence estimates were higher in school-aged and adult groups than in the preschool-aged group; (4) the occurrence estimates were higher in male than in female participants; and (5) stuttering was mild-to-moderate, and secondary behaviors were found when measured.

To identify stuttering, the included studies used various assessment approaches, which were initially developed for the typically developing population, mainly focusing on the identification of speech disfluencies, both with and without frequency cutoff scores. However, no studies included self-reports of experiences related to stuttering. One reason for the heavy focus on stuttering behavior may be the high frequency of older studies included in this review. The multidimensional view of stuttering may represent a more recent understanding of the disorder, one in line with the changes in the diagnostic criteria for stuttering in the latest revision of the International Classification of Diseases (ICD-11, World Health Organization, 2022), to include the significant effects on functioning (e.g., social communication and personal and family life) in addition to observable behaviors. Another reason for the heavy focus on stuttering behavior may be low expectations regarding these individuals’ capability to evaluate and report their own reactions due to reduced language skills (Martin et al., 2009; Næss et al., 2011) and reduced non-verbal mental ability (Næss et al., 2021). However, reactions to stuttering are found in typically developing children from a very low chronological age (Boey et al., 2009), which may correspond to the lower developmental age in individuals with Down syndrome. Furthermore, the existence of affective and cognitive reactions to stuttering has been described in both children (Bray, 2017) and adults (Jackson et al., 2014) with Down syndrome. Based on these indications, individuals with Down syndrome’s own evaluations can be considered in the identification of stuttering.

Although the results of this review showed that listener evaluation in the form of clinical judgment was a common approach, the factors considered to be indicative of stuttering varied across studies, especially those related to types of repetition, as did what threshold disfluencies were considered clinically significant. However, research on typically developing individuals faces the same challenges, which means that comparisons are restricted between both the studies in this review and research projects on stuttering in general (Einarsdottir and Ingham, 2005). The lack of agreement in the field about the indicators of stuttering, as well as at what threshold (cut-off) disfluency is considered stuttering may influence who is considered to need treatment. It may also fuel the discussion about whether the disfluencies seen in individuals with Down syndrome represent genuine stuttering.

In addition to these general challenges within the field of stuttering related to assessment, there are some specific challenges related to the identification of stuttering behaviors in individuals with Down syndrome. As language development is significantly delayed in this population, there are likely differences in the amount of speech material available for listener evaluation across age groups; preschool-aged children with Down syndrome who have begun to speak will, for example, often produce short utterances (Berglund et al., 2001; Zampini and D’Odorico, 2011). This means that the amount of speech material elicited in one speaking situation may be very limited for some participants, as is the case in, e.g., Eggers and Van Eerdenbrugh (2018). As previously mentioned, concerns have been raised regarding the poor reliability of stuttering measures based on the identification of speech disfluencies in speech samples (Cordes, 2000; Einarsdottir and Ingham, 2005; Davidow, 2021), suggesting that the identification of stuttered disfluencies in the typical population can be challenging. Limited speech, in combination with atypical speech features in individuals with Down syndrome, may pose an added challenge in this regard. The reviewed studies that did include inter-rater reliability analysis did, however, report good or very good reliability. The same results were found in Maessen et al. (2023), who included a preselected sample of individuals with Down syndrome who stuttered. Whether the good reliability scores in these studies are related to, for example, speech characteristics, including the frequency of stuttering types, or to the use of summary agreement scores across all disfluency types (i.e., each disagreement has less influence when the number of stuttering disfluencies is large) is unknown.

The abovementioned concerns and limitations related to stuttering assessment, in addition to the quality of the included studies, constitute the frame within which we can interpret and understand the findings of the current review. Nevertheless, studies consistently report high occurrences of stuttering in individuals with Down syndrome when interpreted against the estimated 1% prevalence for the typical adult population (see, e.g., Månsson, 2000) and the 5–11% cumulative incidence of stuttering in typically developing children (Reilly et al., 2009, 2013). The fact that a high percentage of the individuals in the included studies displayed core stuttering behaviors to a degree which they were judged to stutter is in line with previous research, showing that individuals with Down syndrome are vulnerable to speech, language, and communication difficulties (see, e.g., Martin et al., 2009; Næss et al., 2011; Jones et al., 2019; Wilson et al., 2019; Loveall et al., 2021), including stuttering (Kent and Vorperian, 2013). Recent research has also shown that coexisting speech and/or language disorders are common in individuals who stutter and do not have Down syndrome (Wolk and LaSalle, 2023), as are comorbidities between stuttering and other neurodevelopmental disorders (e.g., disorders of intellectual development; Briley and Ellis, 2018). It is therefore likely that the high rate of occurrence is related to an increased vulnerability to stuttering associated with the biomedical condition of Down syndrome and is not a characteristic of Down syndrome.

The occurrence estimates were high (approximately 18–25%) across school-aged and adult participants, while in preschool-aged participants, the occurrence estimate was low relative to the cumulative incidence in typically developing children. These results indicate an opposite pattern to that commonly observed in typically developing individuals, in which the occurrence usually is higher in the preschool years and decreases with age (Bloodstein et al., 2021). Even though the results of the present study may indicate higher occurrences of stuttering in older individuals with Down syndrome, the results do not necessarily mean that the occurrence of stuttering in this population increases with age. As there are no studies following the same participants across time, the results represent the occurrence of stuttering in different age groups and not the developmental pattern of stuttering. The occurrence of stuttering is expected to vary with the sampled age group (Samson, 2022). However, several aspects related to the design of the included studies make it difficult or even impossible to discuss differences in occurrence estimates across age groups. The combination of non-probability sampling techniques, small sample sizes, and age-spread samples do, for example, mean that there are uneven numbers of participants across age groups. Thus, occurrence estimates may suffer from an overrepresentation of age groups where stuttering is more or less common, or findings simply being coincidental as single participants may have a large influence on the results.

A minority of the studies in this review provided information on the occurrence of stuttering by gender. The synthesis of the results of these studies suggests that stuttering was twice as common in male participants as in female participants. However, the gender balance seems to be similar across studies (based on total *n*). This result is in line with findings from studies of the typical adult population, in which more male participants are found to stutter than female participants (gender ratio of between 2:1 and 4:1; Craig et al., 2002). In typically developing young children, the gender distribution is more balanced (Samson, 2022), but more male participants than female participants still stutter (gender ratio of 1.6:1; Sjøstrand, 2022). Whether this asynchrony between genders occurs because of skewed birth figures for boys and girls, because male participants are more vulnerable to stuttering or is related to the indicators of stuttering, is unknown.

Conclusions regarding the characteristics of stuttering are restricted by the specificity of the information provided in the included studies. For example, although studies have consistently reported a high occurrence of stuttering, the current review cannot provide much information regarding the distribution of disfluency types, as the dissertation by Keane (1970) is the only study that provides information about the disfluency types identified in participants who stutter. However, the existence of repetitions, prolongations, and blocks, which are usually included in the evaluation of stuttering in typically developing individuals (Bloodstein et al., 2021), were common identifiers of stuttering across studies. Thus, the results indicate that individuals with Down syndrome display the same speech behaviors as typically developing individuals who stutter.

Furthermore, the focus on the potential adverse effects of stuttering is very limited in the reviewed studies. Even though the participants in the current review exhibited stuttering severity in the mild-to-moderate range, this does not mean that the potential effects of stuttering are not extensive, as studies demonstrated no significant relationship between stuttering frequency and negative feelings about communication (Erickson and Block, 2013).

### 4.1 Implications for practice and research

As stuttering is common, can cause negative reactions (Jackson et al., 2014; Bray, 2017), and have negative consequences for communication in individuals with Down syndrome (Evans, 1977; Maessen et al., 2022), practitioners must refer those with disfluent speech to speech and language therapists for assessment and, eventually, treatment. Thus, validated assessment procedures and research-based treatments developed especially for this population should be trialed in future research. In addition, information that is relevant to teachers, parents, and healthcare professionals should be developed to inform them about the high occurrences of stuttering in individuals with Down syndrome and when referral for the evaluation of stuttering is appropriate.

### 4.2 Limitations

We want to highlight four limitations related to the occurrence estimates of the present study. Because the measures and sample characteristics differ across the studies, it is not straightforward to conclude the occurrence of stuttering. The findings should be interpreted as estimates, as they are likely influenced by (1) sample size, (2) how stuttering is operationalized and assessed, (3) the language proficiency of the participants, and (4) at what age stuttering is measured.

Furthermore, because this review is based on only concurrent data regarding stuttering, we do not know how the occurrence rate by age relates to the tractability of stuttering in this population. For example, it is unknown whether those who stutter at young ages continue to stutter later in life. To answer this question, longitudinal studies are needed. Also, as this review only included studies that investigated the occurrence of stuttering, studies with samples preselected based on fluency status have not been included. This implies that there may be more available research investigating the characteristics of stuttering in individuals with Down syndrome that has not been included in this study. Finally, it should be mentioned that some of the confidence intervals in our inter-rater reliability analysis are wide, indicating a limitation in the precision of these estimates. This uncertainty in some of the estimated effect sizes may reflect the low number of studies in this review (Hazra, 2017), as each disagreement has a large influence on the effect size and its confidence level.

## 5 Conclusion

The results of this systematic review show a high rate of stuttering occurrence in individuals with Down syndrome, independent of assessors, when interpreted against results derived from studies on occurrence estimates in typically developing individuals. This applies to both male and female participants, but the relative proportion of male participants among stutterers is higher. Furthermore, the occurrence in the school-aged and adult participant groups is especially high. While the operationalization of stuttering varied across the studies, the identification of repetitions, prolongations, and blocks was typically included. Stuttering was commonly judged to be mild-to-moderate, and secondary behavior was found when measured.

## Author contributions

SH and K-AN: conceptualization, methodology, formal analysis, resources, investigation, data curation, writing–original draft preparation, and writing–review and editing. SH: visualization and project administration: K-AN and SH: funding acquisition. All authors have read and agreed to the published version of the manuscript.
