# Corrigendum: Stuttering in individuals with Down syndrome: a systematic review of earlier research

**DOI:** 10.3389/fpsyg.2024.1440913

**Published:** 2024-07-03

**Authors:** Silje Hokstad, Kari-Anne B. Næss

**Affiliations:** ^1^Department of Education, Lillehammer, Inland Norway University of Applied Sciences, Lillehammer, Norway; ^2^Department of Special Needs Education, University of Oslo, Oslo, Norway

**Keywords:** Down syndrome, stuttering, speech, disfluency, stuttering assessment, systematic review

In the published article, there was an error in Table 4. The study by Rabensteiner (1975) was missing in the table. The corrected Table 4, “Measurement approaches,” and its caption appear below.

**Table 4:** Measurement approaches.

**Study**	**Assessor(s)**	**Instrument**		
	**Parent(s), SLP(s), author(s)/researcher(s), student(s), stutterer, or other**	**Clinical judgment, parental judgment, self-report, or other**	**Speaking situation as described in study**	**Speech sample (audio/video, duration and number of utterances, words, or syllables), written sources, real-time observation (duration and/or number of utterances, words, or syllables), or own experience**
Devenny and Silverman (1990)	SLPs	Clinical judgment	Conversation about work and recreation	Speech sample (video, 10 min, first 150 words)
Eggers and van Eerdenbrugh (2018)	Authors	Clinical judgment	Play session with toy or book adapted to age and interests	Speech sample (audio, 15 min, 50 utterances^1^)
Gottsleben (1955)	Author and SLPs	Clinical judgment	NR	Written sources
Hokstad et al. (2022)	Researchers	Clinical judgment	Picture book dialogue and story-retelling	Speech sample (audio, unknown duration/number of utterances/words/syllables)
Keane (1970)	1 = SLP 2 = SLPs and other (clinical experience with stutterers)	Clinical judgment	1 and 2 = Interviews about daily life and interests	1 = Real-time observation (ca. 10 min), 2 = Speech sample (video, mean duration 10 min)
Kumin (1994)	Parent	Parental judgment	NR	Real-time observation (NR)
Martyn et al. (1969)	SLPs and students	Clinical judgment	Conversation, interview or reading sample adapted to the level of intellectual disability	Real-time observation (NR)
Preus (1972)	1) NR 2) Other (personnel day institutions) 3) NR	Clinical judgment	1) Spontaneous speech evoked by means of conversation pictures 2) Daily interaction 3) NR	1) Speech sample (audio, mean duration 9.47 min, min/max = 3.5–28 min, minimum 200 words) 2) real-time observation (NR) 3) NR
Rabensteiner (1975)	Other (two observers)	Clinical judgment	Test situation	Real-time observation
Rohovsky (1965)	10 grad. students (speech and hearing science)	Clinical judgment	Story retelling	Speech sample (audio, 30 s)
Salihovic et al. (2012)	SLPs	Clinical judgment	Spontaneous speech elicited through pictures	1 and 2 = Speech sample (audio, minimum 200 syllables) 3 = real-time observation (minimum 200 syllables)
Schieve et al. (2009)	Adult family member (usually parent)	Parental judgment	NR	Real-time observation (NR)
Schlanger and Gottsleben (1957)	Authors/researchers	Clinical judgment	NR	Written sources
Stansfield (1990)	1 = Other (nursing or ATC staff) 2 and 3 = SLP and students	1 = Other (paid caregivers) 2 and 3 = Clinical judgment	1 = NR 2 = Informal interaction 3 = Assessment situation	1 = Real-time observation (NR) 2 = Real-time observation (5 min) 3 = Speech sample (audio, 30 min)

In cases where assessments have been conducted in several stages each stage is numbered, SLP, speech and language pathologist; min, minutes; NR, not reported. ^1^When 50 utterances were not available the maximum number of utterances was used. In cases in which assessments have been conducted in several stages, each stage is numbered, SLP, speech and language pathologist; ATC staff, adult training center staff; min, minutes; NR, not reported; grad., graduate.

The authors apologize for this error and state that this does not change the scientific conclusions of the article in any way. The original article has been updated.

