# Supplementary material for: Stuttering in individuals with Down syndrome: a systematic review of earlier research
Source: Front Psychol. 2023 Nov 29;14:1176743. doi: 10.3389/fpsyg.2023.1176743 (PMC10716236; doi:10.3389/fpsyg.2023.1176743)
Supplement: Supplementary file 1 [file Data_Sheet_1.pdf]

Appendix A. Screening questions

For study selection, we used the following screening questions:

1. Does the source report the occurrence of stuttering in individuals with Down syndrome (number and/or percentage of participants)?

2. Does the source report original individual outcome data on stuttering?

3. Does the source include a sample that is not preselected based on stuttering status?

Heading and abstract screening phase: In cases in which the answer was “No” to one or more of the questions in the screening of the headings and abstracts, the sources were excluded. In cases in which the answer was yes and/or unclear, we retrieved the full text sources for further assessment.

Full-text assessment phase: In cases in which the answer was “Yes” to all three questions, the source was included. In cases in which the answer was “No” to one or more of the questions in the assessment of the full-text sources, the sources were excluded. In cases in which the answers to one or more of the questions were unclear, this was resolved through discussions among the review authors and, on one occasion, through clarification via e-mail correspondence with the study author.

Appendix B. Examples of excluded studies, with reasons

Study	*Reason for exclusion*	*Note*
Buchka (1971)	Source not publicly available.	Failed to retrieve through library or external library services.
Devenny et al. (1990)	Does not report the occurrence of stuttering in individuals with Down syndrome.	Sample is preselected based on fluency status, including only persons who stutter.
Evans (1977)	Does not report the occurrence of stuttering in individuals with Down syndrome.	Reports associations between aspects of speech and language, including features associated with stuttering.
Jones et al. (2019)	Does not report the occurrence of stuttering in individuals with Down syndrome.	Reports occurrence of auditory-perceptual speech features, including features typically associated with stuttering
Maessen, Rombouts et al. (2022)	Does not report the occurrence of stuttering in individuals with Down syndrome.	Sample is preselected based on fluency status, including only people who stutter.
Maessen, Zink et al. (2021)	Does not report the occurrence of stuttering in individuals with Down syndrome.	Sample is preselected based on fluency status, including one group of persons who stutter and one group who do not stutter.
Næss et al. (2021)	Does not report the occurrence of stuttering in individuals with Down syndrome.	Investigates disfluency, not isolated to stuttering.
Otto and Yairi (1974)	Does not report the occurrence of stuttering in individuals with Down syndrome.	Sample is preselected based on fluency status, including only individuals with Down syndrome who do not stutter.
Sommer et al. (2021)	Does not report the occurrence of stuttering in individuals with Down syndrome.	Sample is preselected based on SLP treatment financed via health insurance in Germany.
Van Borsel and Vandermeulen (2008)	Does not report the occurrence of stuttering in individuals with Down syndrome.	Reports the number of individuals with Down syndrome identified with "stuttering-cluttering," not stuttering only.
Willcox (1988)	Does not report the occurrence of stuttering in individuals with Down syndrome.	Sample is preselected based on fluency status. All five participants had been noted to be non-fluent by their speech therapists.

Appendix C. Coding scheme for data extraction

*Only information provided in the research reports were considered (i.e., referenced materials have not been solicited for data extraction).

1. Sample
   1.1. Nationality. Where is the study conducted? If not explicitly stated in the paper, the judgement is based on the affiliation of the first author.
   1.2. Sample size. How many participants with Down syndrome are included in the sample?
   1.3. Gender. How many male and female participants with Down syndrome are included in the sample?
   1.4. Age. (a) What is the mean age of the participants with Down syndrome in years? (b) What are the minimum and maximum ages of the participants in years? Age is rounded to whole numbers (years). In cases in which calculations must be done, we will round to whole numbers.
   1.5. Language proficiency. Is there any information about the participants' language proficiency? In cases in which stuttering is reported for a sub-sample based on language proficiency, the findings for the sub-sample are reported.
2. Measurement approach(es)
   2.1. Assessor. Who evaluated the stuttering; parent/s, SLPs, author/researcher, students, the individual who stutters, or other?
   2.2. Instrument. a) Was stuttering evaluated through clinical judgement (conducted by, for example, SLPs, students, or researchers), parental judgement, self-report, or other? b) In what type of speaking situation was stuttering evaluated (as stated in the paper, if several speaking situations are mentioned but not clearly linked to the stuttering evaluation = NR)? c) Was the data source for the evaluation based on speech samples (*audio/video, duration and/or number of utterances, words, or syllables*), written sources, real-time observation (*duration and/or number of utterances, words, or syllables*), self-reports, or other)?
   2.3. Operationalization. How was stuttering operationalized for the purpose of empirical data collection?
   2.4. Reliability measure. a) What type of inter-rater reliability measure was used for stuttering outcome measures (percentage agreement, correlation coefficient (e.g., Cohen's kappa), other, or no reliability measure)? b) What was the level of agreement between raters?
3. Outcomes
   In cases in which the information we are seeking is not directly reported in the manuscript, this is calculated based on the information provided when possible. In cases in which more than one measure of stuttering is reported, the number of individuals identified as stutterers across the measures is calculated. In cases in which there is more than one time point (i.e., longitudinal studies), the first time point is reported. Outcomes for individuals who stutter must be reported separately (i.e., outcomes for the total sample, including children who do and do not stutter, are not reported).
   3.1. Occurrence.
      3.1.1. What are the percentage and number of participants identified as stuttering?
      3.1.2. Occurrence by gender. What are the percentage and number of males and females identified as stuttering?
      3.1.3. Occurrence by age/age group. a) What is the number of participants identified as stuttering in various age groups (preschool age, school age, adulthood, and mixed age). In cases in which occurrence by age is not specifically reported but the total sample is within a single age group, this should be reported, along with b) the average age of the individuals who stutter (mean and min–max).

3.2. Stuttering behavior.

3.2.1. Frequency of SLDs. a) What is the total frequency of SLDs (mean and minimum/maximum)? What is the frequency of each stuttering type (n of participant displaying each disfluency type, as well as the mean and minimum/maximum for all stuttering participants)?

3.2.2. Stuttering Severity ratings. (a) How severe is the stuttering reported for individuals who stutter (mean and minimum/maximum on the rating scale)? (b) What is the number of participants displaying mild, moderate, or severe stuttering?

3.2.3. Secondary behavior. How many of the participants who stutter display secondary behaviors (% (n))?

3.3. Affective reactions to stuttering. Are outcomes (variable described in methods) related to emotions connected with stuttering (feeling anxious, ashamed, or afraid of talking) reported?

3.4. Cognitive reactions to stuttering. Are outcomes (variable described in methods) related to thoughts (e.g., reports of the anticipation of stuttering and communication attitudes) and identity (e.g., reports of self-confidence) reported?

In studies in which individuals with Down syndrome were part of a larger sample, only the information on the individuals with Down syndrome was coded. In studies in which different groups of individuals with Down syndrome were compared (e.g., individuals living in institutions versus those living at home), information on the total sample was coded. In cases in which the information was not reported, this is marked with NR.

Appendix D. Inter-rater reliability for stuttering variables

Item	Cohen's kappa	SE	*P*	95% CI
Instrument a	.860	.127	< .001	.611, 1.000
Instrument b	1.000	.000	< .001	1.000, 1.000
Instrument c	.860	.127	< .001	.611, 1.000
Operationalization	.860	.127	< .001	.611, 1.000
Occurrence percent	1.000	.000	< .001	1.000, 1.000
Occurrence N	1.000	.000	< .001	1.000, 1.000
Occurrence males percent	.860	.127	< .001	.611, 1.000
Occurrence males N	1.000	.000	< .001	1.000, 1.000
Occurrence females percent	1.000	.000	< .001	1.000, 1.000
Occurrence females N	1.000	.000	< .001	1.000, 1.000
Occurrence by age group	.860	.127	< .001	.611, 1.000
Frequency of SLDs	.860	.127	< .001	.611, 1.000
Frequency of SLD types	1.000	.000	< .001	1.000, 1.000
Stuttering severity ratings	.860	.127	< .001	.611, 1.000
Secondary behavior	1.000	.000	< .001	1.000, 1.000
Affective reactions	.724	.157	< .001	.416, 1.000
Cognitive reactions	.860	.127	< .001	.611, 1.000
Inter-rater reliability	1.000	.000	< .001	1.000, 1.000

Note. n = eight studies. Disagreements range from 0 – 2 per item.

Appendix E. Inter-rater reliability for Quality appraisal

Item	Cohen's kappa	SE	*p*	95% CI
1	.877	.113	< .001	.656, 1.000
2	.877	.113	< .001	.656, 1.000
3	1.000	.000	< .001	1.000, 1.000
4	1.000	.000	< .001	1.000, 1.000
5	.877	.113	< .001	.656, 1.000
6	.877	.113	< .001	.656, 1.000
7	.757	.143	< .001	.477, 1.000
8	.877	.113	< .001	.656, 1.000
9	1.000	.000	< .001	1.000, 1.000
10	.753	.150	< .001	0.459, 1.000
11	.630	.170	< .001	.297, .963
12	.630	.170	< .001	.297, .963
13	.877	.113	< .001	.656, 1.000
14	1.000	.000	< .001	1.000, 1.000
15	.753	.150	< .001	0.459, 1.000
16	1.000	.000	< .001	1.000, 1.000
17	.877	.113	< .001	.656, 1.000
18	.877	.113	< .001	.656, 1.000
19	1.000	.000	< .001	1.000, 1.000
20	.877	.113	< .001	.656, 1.000

Note. n = nine studies. Disagreements range from 0 – 3 per item.

Appendix F. Operationalization of stuttering

Study	Operationalization
Devenny and Silverman (1990)	Existence of involuntary repetitions and prolongations, effort in overcoming blocks, and secondary behaviour.
Eggers and van Eerdenbrugh (2018)	Three stuttering-like disfluencies (part-word repetition, single-syllable word repetition, prolongation, block, and broken word) or more per 100 syllables.
Gottsleben (1955)	NR.
Hokstad et al. (2022)[1]	1) Stuttering-like disfluencies (repetition of sounds, syllables, or monosyllabic words, prolongations of sounds, and blocks).<br>Or<br>2) *The Stuttering Severity Rating Scale (SSR):* Perceptual rating of stuttering severity on a 10-point scale (0–9), on which 0 = no stuttering, 1 = extremely mild stuttering, and 9 = extremely severe stuttering.<br>Participants were grouped based on whether they had %SLD above 0% or at or above 3% or an SSR > 0[1].
Keane (1970)	1) Three or more disfluencies (SLDs and ODs).<br>2) Stuttering refers to any one or combination of the following criteria: A) frequently occurring or distinctive sound, syllable, and/or one-syllable-word repetitions, sound prolongations, and/or involuntary hesitations in speech behaviour that appear not to be readily controllable, B) any visible and/or audible evidence of struggle behaviour in the form of forcing, blocking, unusual stress or tension, facial grimaces, bodily movements made in conjunction with an attempt to initiate or force sound production, and/or reactions of anticipation or avoidance when producing certain sounds and/or syllables in the form of any device employed to postpone, disguise, start, or release the sound or syllable (e.g., in the form of repetitions of words or phrases), C) any other consistent or distinctive breaks, disturbances, or interruptions in the forward flow of speech that call attention to themselves and may interfere with communication, and D) changes in rate, pitch, inflectional patterns, loudness, articulation, and/or vocal quality that accompany a so-called "stuttering moment."<br>3) Agreement among two of three judges.
Kumin (1994)	NR.
Martyn et al. (1969)	Showing symptoms within one or more groups of symptoms to a degree that sets an individual off from the remainder of the population. Stuttering symptoms:1) blocks, repetitions, prolongations, stickings, grimaces, forcings, or other rhythm brakes or interruptions in the forward flow of speech, 2) fear or anticipation of blocking, fear of the inability to speak, or related symptoms prior to words or speaking situations, and 3) a self-concept that includes a picture of oneself as a stutterer, a stammerer, a speech blocker, or a person lacking normal speech fluency.

Note. [1]While no diagnostic evaluation was undertaken in this study, the authors state that a %SLD at or above 3% and an SSR > 0 are regarded as indicative of stuttering according to the literature.

Appendix F. (continued)

Study	Operationalization
Preus (1972)	1a) Five stuttering symptoms (whole-word repetitions, part-word repetitions, and prolongations) per 100 words.<br>Or<br>1b) Five stuttering symptoms per 100 words, excluding whole-word repetitions.<br>2) Individual stutters often (4) or nearly always (5) on a 5-point rating scale.<br>3) Secondary symptoms (e.g., body movements, postponement, and avoidance).
Rabensteiner (1975)	NR.
Rohovsky (1965)	Existence of blockings; prolongations; repetitions of words, syllables, sounds, or mouth postures, all of which produce interruptions. Stuttering severity rating on a scale from 1 to 5 (1 = mild stuttering, 3 = moderate stuttering, 5 = severe stuttering).
Salihovic et al. (2012)	SSI: (1) Frequency of stuttering, (2) duration of stuttering blocks, (3) physical concomitants, and (4) tables summarizing the degree of stuttering (mild, moderate, severe, or very severe).
Schieve et al. (2009)	NR.
Schlanger and Gottsleben (1957)	Clonic stuttering (repetitions), tonic stuttering (blocking, interruptions), and secondary reactions.
Stansfield (1990)	The stuttering interview – total number of words spoken; the number, percentage, and type of dysfluent words; and severity of the dysfluency, following SSI and SIA procedures.

Note. NR = not reported, SSR = The Stuttering Severity Rating Scale (Onslow et al., 2020), SSI = The Stuttering Severity Instrument (Riley, 1980; 1994), SIA = The Stuttering Interview, Form A (Ryan, 1974).

References

*Included in review

Buchka, M. (1971). The language of the mongoloid child. Heilpadagogik. 40, 308-312. http://ovidsp.ovid.com/ovidweb.cgi?T=JSandPAGE=referenceandD=psyc2andNEWS=NandAN=1972-29545-001

*Devenny, D. A., and Silverman, W. P. (1990). Speech dysfluency and manual specialization in Down's syndrome. J. Intellect. Disabil. Res. 34, 253-260. https://doi.org/10.1111/j.1365-2788.1990.tb01536.x

Devenny, D. A., Silverman, W., Balgley, H., Wall, M. J., Sidtis, J. J. (1990). Specific motor abilities associated with speech fluency in down's syndrome. Journal of Mental Deficiency Research. 34, 437-43. https://doi.org/10.1111/j.1365-2788.1990.tb01554.x

*Eggers, K., and Van Eerdenbrugh, S. (2018). Speech disfluencies in children with Down Syndrome. J. Commun. Disord. 71, 72-84. https://doi.org/10.1016/j.jcomdis.2017.11.001

Evans, D. (1977). The development of language abilities in mongols: a correlational study. Journal of Mental Deficiency Research. 21, 103-117.

*Gottsleben, R. H. (1955). The incidence of stuttering in a group of mongoloids. Training School Bulletin. 51, 209-218.

*Hokstad, S., Næss, K. A. B., Yaruss, J. S., Hoff, K., Melle, A. H., and Lervåg, A. O. (2022). Stuttering behavior in a national age cohort of Norwegian first-graders with Down syndrome. J. Speech Lang. Hear. 65:11. https://doi.org/10.1044/2022_JSLHR-21-00605

Jones, H. N., Crisp, K. D., Kuchibhatla, M., Mahler, L., Risoli Jr, T., Jones, C. W., and Kishnani, P. (2019). Auditory-perceptual speech features in children with Down syndrome. Am. J. Intellect. Dev. Disabil. 124:4. https://doi.org/10.1352/1944-7558-124.4.324

*Keane, V. A. (1970). An investigation of disfluent speech behavior in Down's syndrome [Unpublished dissertation]. [Eugene (Oregon)]: University of Oregon.

*Kumin, L. (1994). Intelligibility of speech in children with Down syndrome in natural settings: Parents' perspective. Perceptual and Motor skills. 78, 307-313.

Maessen, Rombouts, E., Maes, B., and Zink, I. (2022). Influence of gestures on the intelligibility and comprehensibility of utterances with stuttering events in individuals with Down syndrome. J. Commun. Disord. 95, 106178–106178. https://doi.org/10.1016/j.jcomdis.2021.106178

Maessen, B., Zink, I., Maes, B., and Rombouts, E. (2021). An experiment on measuring awareness of stuttering in individuals with Down syndrome. J. Fluen. Disord. 68, 105849. https://doi.org/10.1016/j.jfludis.2021.105849

*Martyn, M. M., Sheehan, J. and Slutz, K. (1969). Incidence of stuttering and other speech disorders among the retarded. Amer J Ment Deficiency.

Næss, K. A. B., Nygaard, E., Hofslundsengen, H., & Yaruss, J. S. (2021). The association between difficulties with speech fluency and language skills in a national age cohort of children

with Down syndrome. *Brain Sciences*, *11*(6), 704. https://doi.org/10.3390/brainsci11060704

Onslow, M., Webber, M., Harrison, E., Arnott, S., Bridgman, K., Carey, B., Sheedy, S., O'Brian, S., MacMillan, V., and Lloyd, W. (2020). The Lidcombe program treatment guide. Lidcombe Program Trainers Consortium. https://www.uts.edu.au/sites/default/files/2020-07/Lidcombe%20Program%20Treatment%20Guide%202020%201.0_1.pdf

Otto, F. M., and Yairi, E. (1974). An analysis of speech disfluencies in Down's syndrome and in normally intelligent subjects. J. Fluen. Disord. 1:4, 26-32. https://doi.org/10.1016/S0094-730X(74)80024-0

*Preus, A. (1972). Stuttering in Down's syndrome. Scandinavian Journal of Educational Research. 16, 89-104.

*Rabensteiner, B. (1975). [Social behavior, musicality, and visual perception in mongoloid children (author's transl)]. Padiatrie Und Padologie. Supplementum. 4, 59-69. http://ovidsp.ovid.com/ovidweb.cgi?T=JSandPAGE=referenceandD=med1andNEWS=NandAN=127148

Riley G.D. (1980) Stuttering Severity Instrument. CC Publications, Oregon.

Riley G. D. (1994). Stuttering severity instrument for children and adults, Third Edition. Examiner's manual and picture plates. Pro-ed Inc.

*Rohovsky, K. A. (1965). A study of stuttering in institutional and non-institutional mongoloids [Unpublished master's thesis]. [Columbus]: Ohio State University.

Ryan B.P. (1974) Programmed Stuttering Therapy for Children and Adults. Charles C. Thomas

*Salihovic, N., Hasanbasic, S., and Begic, L. (2012). Incidence of Stuttering in School-Age Children with Down Syndrome. The Journal of Special Education and Rehabilitation. 13:1/2, 31.

*Schieve, L. A., Boulet, S. L., Boyle, C., Rasmussen, S. A., and Schendel, D. (2009). Health of children 3 to 17 years of age with Down syndrome in the 1997-2005 National Health Interview Survey. Pediatrics (Evanston). 123:2. https://doi.org/10.1542/peds.2008-1440

*Schlanger, B. B., and Gottsleben, R. H. (1957). Analysis of speech defects among the institutionalized mentally retarded. Journal of Speech and Hearing Disorders. 22, 98-103.

Sommer, M., Waltersbacher, A., Schlotmann, A., Schröder, H., and Strzelczyk, A. (2021). Prevalence and therapy rates for stuttering, cluttering, and developmental disorders of speech and language: evaluation of German health insurance data. Front. Hum. Neurosci. 15, 176. https://doi.org/10.3389/fnhum.2021.645292

*Stansfield, J. (1990). Prevalence of stuttering and cluttering in adults with mental handicaps. Journal of Intellectual Disability Research. 34, 287-307. https://doi.org/10.1111/j.1365-2788.1990.tb01541.x

Van Borsel, and Vandermeulen, A. (2008). Cluttering in Down syndrome. Folia Phoniatrica Logopaedica. 60:6, 312-317. https://doi.org/10.1159/000170081

Wilcox, A. (1988). An investigation into non-fluency in Down's syndrome. Br. J. Disord. Commun. 23, 153-170. https://doi.org/10.3109/13682828809019884
